# AI-based optimization for US-guided radiation therapy of the prostate

**DOI:** 10.1007/s11548-022-02664-6

**Published:** 2022-05-20

**Authors:** Stefan Gerlach, Theresa Hofmann, Christoph Fürweger, Alexander Schlaefer

**Affiliations:** 1 Institute of Medical Technology and Intelligent Systems, Hamburg University of Technology, Hamburg, Germany; 2 Europäisches Radiochirurgie Zentrum München, Munich, Germany; 3 Department for Stereotaxy and Functional Neurosurgery, University Hospital of Cologne, Cologne, Germany

**Keywords:** Robotic ultrasound, Heuristic optimization, Treatment planning, Convolutional neural network, Simulated annealing

## Abstract

**Objectives:**

Fast volumetric ultrasound presents an interesting modality for continuous and real-time intra-fractional target tracking in radiation therapy of lesions in the abdomen. However, the placement of the ultrasound probe close to the target structures leads to blocking some beam directions.

**Methods:**

To handle the combinatorial complexity of searching for the ultrasound-robot pose and the subset of optimal treatment beams, we combine CNN-based candidate beam selection with simulated annealing for setup optimization of the ultrasound robot, and linear optimization for treatment plan optimization into an AI-based approach. For 50 prostate cases previously treated with the CyberKnife, we study setup and treatment plan optimization when including robotic ultrasound guidance.

**Results:**

The CNN-based search substantially outperforms previous randomized heuristics, increasing coverage from 93.66 to 97.20% on average. Moreover, in some cases the total MU was also reduced, particularly for smaller target volumes. Results after AI-based optimization are similar for treatment plans with and without beam blocking due to ultrasound guidance.

**Conclusions:**

AI-based optimization allows for fast and effective search for configurations for robotic ultrasound-guided radiation therapy. The negative impact of the ultrasound robot on the plan quality can successfully be mitigated resulting only in minor differences.

## Introduction

Robotic radiation therapy allows for flexible shaping of the dose gradient to very precisely approximate the target, while avoiding critical surrounding tissue. Furthermore, target movement, e.g., due to breathing, can be compensated for during treatment [1]. However, to achieve this precision and motion compensation, the position and shape of the target need to be tracked during treatment. In clinical practice, e.g., using the CyberKnife system, the target is usually tracked by periodically observing the target’s position using X-ray images either by implanting fiducials or by estimating the position directly from the X-ray images [1]. To allow for a higher temporal resolution, the target motion can be correlated with the breathing motion which is tracked using optical cameras [1].

However, this approach has several disadvantages. First, implanting fiducials has associated risks for complications [2]. Furthermore, correlating breathing motion with the actual target motion can introduce additional errors and deformation is not considered [1]. Additionally, conventional intra-fraction imaging relies on ionizing radiation which can be avoided with different tracking methods.

Recently, several noninvasive volumetric tracking methods based on non-ionizing imaging have been proposed including magnetic resonance imaging (MRI) [3–6] and ultrasound (US) [7, 8]-based methods. While MRI presents an interesting modality for precise tracking, MR-Linacs come at a high initial cost. For intra-fraction tracking, fast, volumetric robotic US is a promising approach [9–11] since it allows to monitor the target over an extended period of time while maintaining sufficient image quality.

A challenge when integrating robotic US guidance is the proximity of the US transducer to the target which can block beams due to its radio-opaqueness [12]. While there are advances for implementing a (approximated) radiolucent US setup [13], they require specialized hardware and the remaining influence on the beam delivery still has to be accounted for. In general, not all beam directions are equally useful for an effective treatment, i.e., some beam directions will not be used after optimization. Hence, the US robot should ideally block mostly unused beam directions while having minimal effect on the beams used for treatment. Unfortunately, it is not straightforward to predict which beams will be useful. Typically, one needs to solve the inverse planning problem to analyze which beam directions are used less often. Moreover, the inverse planning problem is degenerate in that usually a large number of different treatment beam sets with very similar objective values exist, i.e., knowing one solution does not imply that no other solution with similar dose distribution but different unused beam directions exists. Considering the combinatorial nature of the problems to find a subset of all possible beams as the treatment beams and one of the many possible US-robot positions and configurations, we previously used a single solution and the resulting beam set to guide the search for a good US-robot position and configuration [14].

Machine learning approaches for estimation of useful beam directions have been proposed [15–17]. We study a convolutional neural network (CNN) for evaluating beam directions to overcome the limitation that a single solution does not indicate whether other solutions may exist. The CNN is trained on a large set of different treatment plans and hence resembles a heuristic that should give a more inclusive evaluation whether blocking certain beam directions will have a negative impact. A key advantage is the very fast evaluation of possible beams [16], as no treatment planning has to be done. We refer to the integration of different AI methods, i.e., machine learning, heuristic search, and deterministic optimization as AI-based optimization.

We give details on the CNN, the training, and the integrated search for US-robot position and configurations. Our results on actual prostate cancer treatment cases illustrate that the CNN-based heuristics can effectively guide beam selection and that the integration with the US-robot pose optimization leads to a plan quality comparable to plans without US guidance.

## Material and methods

### Dose optimization and patient data

We generate treatment plans based on computed tomography (CT) scans of 50 patients previously treated for prostate cancer. First, prostate, bladder, and rectum are delineated and labeled as planning target volume (PTV) and organs at risk (OARs), respectively. Then, additional SHELL structures are introduced around the PTV at 3 mm and 9 mm distance to control for dose in normal tissue.

We discretize PTV, OARs, and SHELLs with a resolution of $3 \times 3 \times 3$ mm and optimize the coverage of the PTV, i.e., the proportion of the PTV that receives at least the prescribed dose, by minimizing the underdosage of the PTV. We model this optimization problem as a linear programming problem with hard constraints on the maximum dose of PTV, OARs, and SHELLs. The optimization problem is solved with respect to a set of candidate beams using our in-house planning framework [18]. This results in a small subset of weighted beams that are optimal with respect to the given set of candidate beams. The weight of the beams is the activation time (MU) of the respective beam in the resulting treatment plan.

During treatment plan generation, multiple clinical goals can be considered besides coverage. A pareto-optimal plan cannot be improved with respect to one goal without impairing another. Linear programming allows step-wise optimization of every individual goal to generate a pareto-optimal treatment plan [18]. In this study, we first optimize PTV coverage. Second, we optimize total MU of all beams, while fixing lower bounds to the dose on the voxels in the PTV. Thereby, we can potentially decrease total MU while not decreasing coverage when treatment plans allow for further improvement after the first optimization step.

For our experiments, we consider a prescribed dose of 36.25 Gy for the PTV and maximum doses for the PTV and OARs of 40.25 Gy and 36 Gy, respectively. We tune the maximum dose to the SHELL for each patient such that the coverage of the PTV is 95% when no US robot is used. We fix these constraints for all experiments so that the effect of the optimization strategies for US robot can be compared directly.

We sample 6000 candidate beams with IRIS collimators [19] with diameters of 10, 15, 20, 30, and 40 mm and constrain each beam’s activation time by 300 MU and the total activation time by 40,000 MU.

### Robotic ultrasound setup

**Fig. 1:**
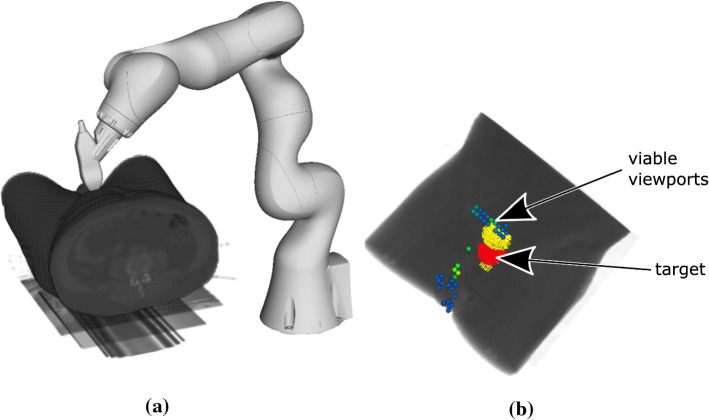
Setup for robotic US guidance on the left (**a**) and example for distribution of viable viewports on the right (**b**). On the right, the target is highlighted in red, the OARs in yellow. Color of viewports corresponds to the maximum HU on the respective path—blue lower, green higher

Figure 1a shows the general setup. The US transducer is mounted to a light-weight 7 degrees-of-freedom (DoF) robot (KUKA LBR Med 7) which is certified for human–robot collaboration. While our approach can be adapted to any suitable US probe, we consider the matrix probe 3V-D (GE Healthcare, Chicago, USA) with a footprint of 24$\times$26 mm, 1.5–4.0 MHz frequency and 90-degree field of view. Naturally, the optimal setup of the US robot with respect to minimizing the impact on the plan quality is highly dependent on the position of the US transducer since it is usually the closest beam-blocking part to the target. For intra-fraction tracking, the US transducer must be positioned to have the target in the field of view. Furthermore, the target must not be obstructed by bones. Similarly, gas-filled cavities inside the body prevent acoustic coupling and must be avoided for sufficient image quality. Additionally, we consider constraints on the maximum penetration depth for the US echo.

We estimate feasible viewports on the discretized surface of the skin considering the CT gray scale values *v* along a line between the skin point and the target center. We exclude viewports with $\max (v) > 1300 \text { HU}$ (dense tissue) and $\min (v) < 400 \text { HU}$ (gas) and also restrict the maximum distance through the tissue to 120 mm. An example of a map of feasible viewports is shown in Fig. 1b. By directing the transducer’s axial axis at the centroid of the target, we assume that feasible viewports allow for enough visibility of the target to allow for tracking. The last rotational DoF of the transducer is defined such that the mounting points towards the base of the US robot to decrease the search space while allowing enough kinematic flexibility for the configuration of the US robot. Furthermore, the 7. DoF of the US robot (LIFT angle) is also included as a decision variable. Additionally, the LIFT angle allows the US robot to move during treatment without changing the pose of the US transducer. Therefore, we study the effect of including three LIFT angles for each robot position while only consider a beam blocked if it is blocked by all resulting US-robot configurations.

**Fig. 2:**
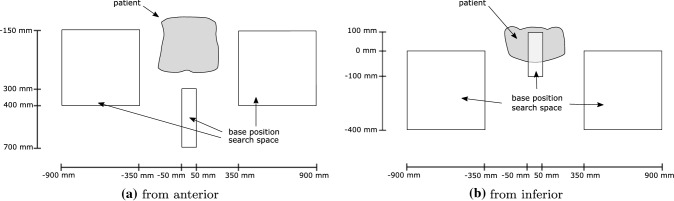
The considered search space for the US-robot’s base position

The robot’s base can be positioned anywhere beside the patient or between the patient’s legs such that it does not collide with the patient and other medical equipment. We restrict the search space for the US-robot’s base position to boxes beside the patient and between the patient’s legs (Fig. 2) with a fixed rotation to reduce the search space. Since the geometry of the robot is known, we can determine whether a specific candidate beam is blocked by the robot or US transducer using a projection-based approach [20]. We consider transducer and target motion due to breathing by adding an extra margin of 20 mm to the projection of robot and transducer.

### CNN-based candidate beam generation

In this study, we extend our approach for candidate beam generation [16] and use it both for guiding the robot pose optimization and for treatment plan generation.

**Fig. 3:**
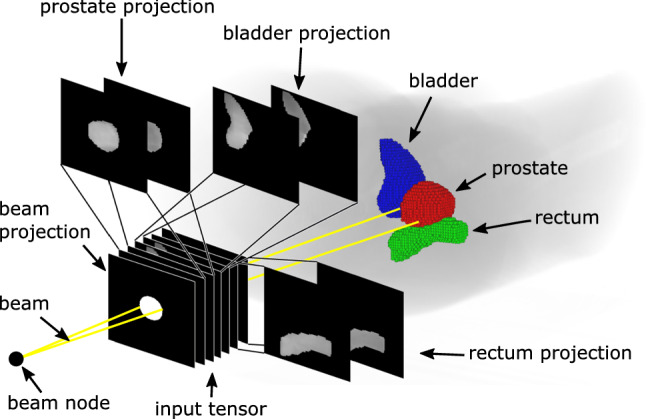
Feature generation per beam. The input tensor consists of a beam projection and minimum and maximum radiological depth projections of PTV and OARs on the same plane. Note that the actual projection plane is located at the PTV’s centroid

We predict the quality, i.e., the activation time, of each candidate beam independently using a CNN based on DenseNet-121 [21] with pretrained weights from ImageNet [22]. We concatenate 7 images for each beam as input to the CNN (Fig. 3). These images are projections on the plane which is perpendicular to the line between the beam’s origin and the PTV’s centroid and located at the PTV’s centroid. The pixel size corresponds to 1$\times$1 mm at the plane and images span 150$\times$150 pixels. The first image is a binary image encoding the location and size of the beam influence relative to the relevant organ structures. The remaining images are the projections of the PTV and the two OARs. Here, we encode the minimum and maximum radiological depth in the images to relay volumetric information to the CNN. Note that these images contain information about the distance to the beam source as well as the radiological properties of the tissue. Starting with the DenseNet architecture, we adjust the kernel depth of the first convolutional layer to allow for a 7-channel input and copy the pretrained weights of the original first convolutional layer in the extended kernel. The last fully connected layer is replaced by a fully connected layer with one output and initialized with random weights.

**Fig. 4:**
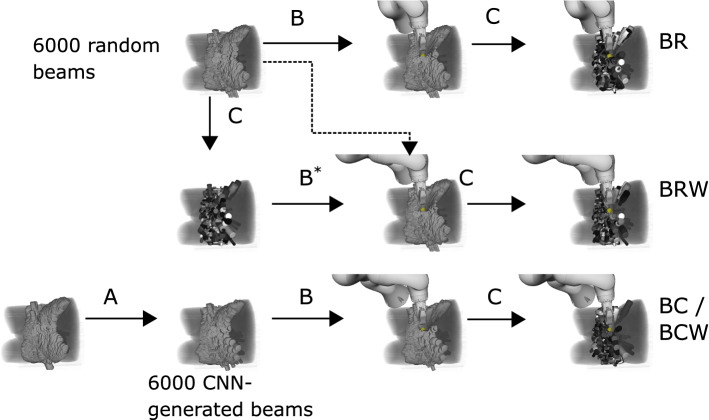
Flowchart of treatment plan generation with the presented objective functions for optimization of robot and probe setup. In each panel, the resulting set of beams and robot setup is shown, representing the state of the optimization problem. A: CNN sampling; B: heuristic US-robot setup optimization; C: treatment plan optimization; $\text {B}^*$: uses the weighted beam set for pose search but applies the found pose to the original beam set. For beam sets with weight, lighter color corresponds to higher beam weight

**Fig. 5:**
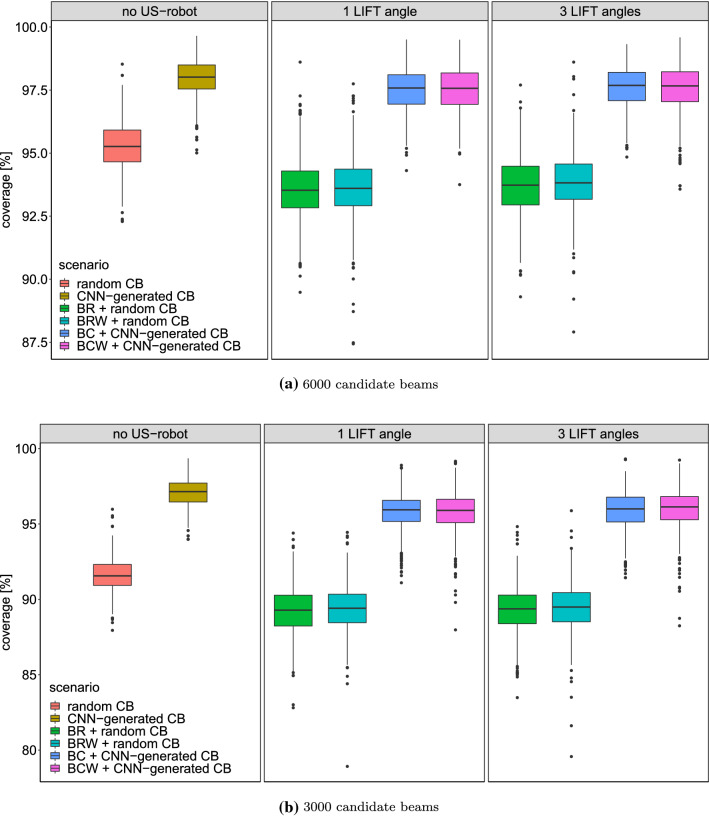
Boxplot of the coverage for reference treatment plans with no US robot, optimized US-robot setup utilizing 1 LIFT angle, and utilizing 3 LIFT angles for 6000 candidate beams (**a**) and 3000 candidate beams (**b**). The same candidate beam set was used for setup optimization and treatment plan generation. RCB: random candidate beams; CNN-CB: CNN-generated candidate beams; BR, BRW, BC, BCW: respective optimization function

The CNN is trained on treatment plans computed using the conventional approach with candidate beams sampled from a random heuristic. Since there is an imbalance in the training data of more unweighted than weighted beams, $w_b \approx 19$, we leave out unweighted beams during training with a probability of $p_b = 1/w_b$. We use threefold cross-validation for training where we split our set of 50 patients into three groups, where each patient occurs in one group. The groups are split such that the distribution of PTV sizes is similar in each group. We train on two groups and evaluate on the remaining group, respectively. Treatment plans for training are generated 30 different candidate beam sets for each patient, resulting in $30\cdot 50 = 1500$ beam sets with 6000 beams each. We run the training for 15 epochs using Adam optimizer [23] with $\beta _1 = 0.9$, $\beta _2=0.999$, and $\epsilon = 10^{-7}$ and decrease the learning rate from $10^{-3}$ to $10^{-5}$ by a factor of 10 every 5 epochs. We forego extensive hyperparameter optimization due to the large computational effort which a complete evaluation of the CNN including beam generation and US-robot pose optimization would mean for each set of hyperparameters. During training, we use the following loss function

$$\begin{aligned} l = {\left\{ \begin{array}{ll} \left( w_{\mathrm {p}} - w_{\mathrm {g}}\right) ^2 \cdot \dfrac{c_{\mathrm {des}}}{c_{\mathrm {p}}}, &{} \quad \text {for }\; w_{\mathrm {g}} = 0\\ \\ \left( w_{\mathrm {p}} - w_{\mathrm {g}}\right) ^2 \cdot \dfrac{c_{\mathrm {p}}}{c_{\mathrm {des}}}, &{} \quad \text {for} \; w_{\mathrm {g}} > 0\end{array}\right. } \end{aligned}$$

where $w_{\mathrm {p}}$ is the predicted, $w_{\mathrm {g}}$ is the ground truth beam weight, $c_{\mathrm {des}} = 95\%$ is the target coverage, while $c_{\mathrm {p}}$ is the coverage of the treatment plan for the respective beam. While constraints are constant for each different beam set, these treatment plans are generated automatically and can exhibit differences in coverage. The weighting aims to respect this difference during training. It expresses that weighted beams of treatment plans with high coverage are more likely to be useful, while unweighted beams of low coverage workflows are more likely to be even less useful. Note that by using a constrained optimization for treatment plan optimization, upper dose constraints are not violated in any generated treatment plan.

Candidate beams for new patients are then generated by selecting randomly sampled beams based on the CNN-predicted beam’s weight $w_{\mathrm {p}}$. A beam is selected with the probability $p = \frac{w_{\mathrm {p}}}{w_{\max }}$, where $w_{\max } = 300$ is the maximum weight per beam. New candidate beams are generated until the specified number of sampled beams has been selected. The candidate beams are then optimized by the same linear program which is used with conventionally generated candidate beams.

### Optimization of robot and probe setup

Since simultaneous optimization of dose and US-robot setup would be an infeasible combinatorial problem given the large search space, we solve both independently using a heuristic search algorithm to optimize the US-robot setup. We approximate the impact of a US-robot setup on the search space by the available candidate beams. However, not all beams are equally useful for generating clinically acceptable treatment plans. Since evaluation of the inverse problem for every US-robot pose is infeasible, we utilize a CNN to estimate the quality of a candidate beam. We compare four optimization functions:

- The number of all blocked randomly sampled candidate beams (**BR**).
- The total sum of the blocked randomly sampled beams’ weight (**BRW**). Here, the weight corresponds to the weight after treatment plan computation without the US robot. Therefore, only actually weighted beams influence the optimization value.
- The number of all blocked CNN-generated beams (**BC**) which reflects the idea to position to US robot such that it covers predominantly unused portions of the available space of candidate beams.
- The total sum of predicted weight of all blocked CNN-generated beams provides a more continuous estimate of useful beam directions. Therefore, we study the blocked CNN-generated beam’s weight (**BCW**) as an optimization function which may provide a smother gradient compared to BC.

Figure 4 shows how we use these objective functions to generate treatment plans. The CNN-based optimization functions are essentially an approximation of the resulting beam set from evaluation of the inverse problem with multiple candidate beam sets. We refer to the last two functions as AI-guided, as they integrate different AI methods, i.e., machine learning, heuristic search, and deterministic optimization.

We use simulated annealing (SA) to optimize the US-robot setup and optimize the state containing the robot base position, the LIFT angle(s), and the US transducer position. Initially, the temperature for the SA algorithm, determining the probability of accepting a worst state as a successor, is set to $\Theta _0=10$ and halved every 100 iterations. We iterate 1000 times. Since the algorithm is heuristic and the solution also depends on the initial state, we repeat the optimization with five random initial states and consider the best state found with respect to the objective function.

## Results

We repeated treatment plan generation with 10 different candidate beam sets for each case to decrease the influence of randomness in candidate beam generation. In total we generated 88 560 treatment plans for evaluation. We use the Wilcoxon rank sum test for significance tests and apply a 0.05 cutoff for rejecting the null-hypothesis that two distributions are the same. Note that we evaluate the dose on the same grid that we used for optimization. This makes generated treatment plans more directly comparable since it reduces the effects of discretization. The resulting coverage when generating treatment plans without a US robot and with the introduced optimization approaches are shown in Fig. 5a. Here, the same beam sets are used for US-setup optimization and treatment plan generation. Both BR and BW cannot compensate completely for the impact of the US robot on the coverage. The mean difference between 6000 random generated candidate beams with no US robot (95.31%) and BW with 3 LIFT angles (93.66%) is significant when considering a Wilcoxon rank sum test ($p = 2.4e^{-108}$).

**Table 1:** Mean resulting objective value and standard deviation from US-setup search for all patients

	BR	BRW	BC	BCW
1 LIFT	645 ± 234	6045 ± 1605	912 ± 283	6350 ± 1505
3 LIFT	643 ± 226	5665 ± 1443	907 ± 281	5613 ± 1614
*p* value	0.962	0.000646	0.828	4.38e$-$11

*p* values for difference between using one and three LIFT angles are shown for each objective function with respect to the Wilcoxon ranks sum test

**Fig. 6:**
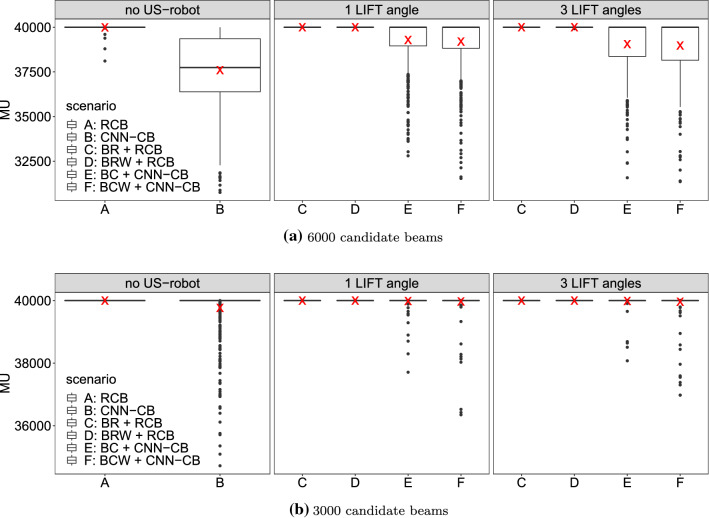
Boxplot of MU after MU optimization. Treatment plans are shown with optimized US-robot setup and without US robot for 6000 (**a**) and 3000 (**b**) candidate beams. The red cross represents the mean MU. RCB: random candidate beams; CNN-CB: CNN-generated candidate beams; BR, BRW, BC, BCW: respective optimization function

However, using 6000 CNN-generated candidate beams can more than offset the negative impact of the US robot on the coverage compared to randomized candidate beams when using 1 LIFT angle or 3 LIFT angles (97.14%, $p = 2.1e^{-143}$; 97.20%, $p = 2.1e^{-149}$). The differences in mean coverage when using 1 or 3 LIFT angles are minor for BR (93.58–93.66%; $p = 0.28$) and for BW (93.61–93.86%;$p = 1.0e^{-4}$), respectively. It does not increase significantly for BC (97.13–97.23%; $p = 0.12$) and BCW (97.15–97.20%; $p = 0.14$).

Even when using only 3000 CNN-generated candidate beams, average coverage is still over 95% while considering the US robot as indicated by Fig. 5b. Difference in average coverage for 3000 CNN-generated candidate beams between no US robot and BCW with three LIFT angles is 0.97 pp ($p = 3.7e^{-64}$).

Table 1 shows the mean objective value using the different optimization strategies. Note that relative differences are larger and significant for the weighted optimization functions between using one and three LIFT angles.

With 6000 CNN-generated candidate beams, full coverage can often be achieved with generated treatment plans. Therefore, we evaluate the remaining potential for treatment plan improvement by optimizing total MU after coverage optimization. Here, minimum bounds on PTV voxels are equal to the dose after coverage optimization allowing us to minimize MU while not affecting coverage. Figure 6 shows the resulting optimized total MU while preserving the coverage shown in Fig. 5. When using 6000 CNN-generated candidate beams, treatment plans can be further optimized as shown in Fig. 6a. In contrast, for 3000 candidate beams only few treatment plans can be improved. Therefore, only outliers are shown below 40,000 MU.

**Fig. 7:**
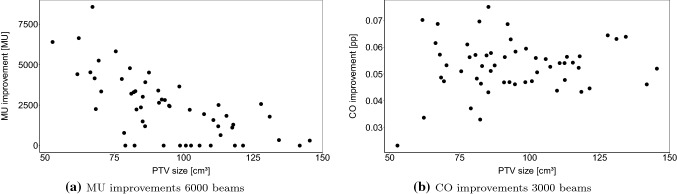
Average total MU improvements (**a**) with respect to PTV size of treatment plans with 6000 CNN-generated candidate beams over 6000 randomly sampled candidate beams without a US robot. In comparison, coverage improvements with 3000 beams are shown (**b**). Each dot represents the average of 10 different candidate beam sets

Still, MU improvements display a wide range. Figure 7a shows the dependence of MU improvements on the patient’s PTV size. The size correlates with the MU improvement with the Spearman’s correlation coefficient $r_s=-0.68$. When discounting for treatment plans which did not achieve full coverage and therefore have no improvement in total MU, then $r_s=-0.73$. In contrast, as Fig. 7b illustrates, that coverage improvements do not show this relationship for 3000 beams. Note that we show the result for 3000 beams here since few treatment plans could achieve full coverage and total MU could rarely be decreased as Fig. 6b shows. Therefore, most are pareto-optimal and can be compared directly in terms of coverage.

## Discussion

We have shown a combination of different AI methods, i.e., machine learning, heuristic search, and deterministic optimization, for optimizing the setup of robotic US guidance in radiation therapy. This idea is similar to other approaches for solving combinatorial problems with machine learning [24]. Considering Fig. 5, we have shown that combining CNN-generated candidate beams with optimization of the US-robot setup can improve treatment plan quality and compensate for the negative impact of the US robot. Since full coverage can often be achieved with 6000 CNN-generated candidate beams, average difference in coverage when the US robot is present is small compared to no US robot.

For 3000 beams, coverage is still over the targeted 95% coverage even when considering the US robot. While the decrease in coverage when considering the US robot is small, it is still significant. Note that most plans could not be improved after coverage optimization with 3000 candidate beams as shown in Fig. 6b.

Subsequent MU optimization can decrease total MU for treatment plans with 6000 CNN-generated candidate beams even when using the US robot. However, subsequent MU optimization shows that impact of the US robot is still significant but can be reduced by optimization of the setup. Note that treatment plans which were used for training of the CNN were only optimized for coverage. Therefore, beams selected by the CNN-based approach are biased towards generating treatment plans with high coverage. Training a CNN on MU optimized treatment plans and combining beam sets from both CNNs may improve total MU further, especially for larger PTVs where MU improvements are low, as Fig. 7a shows. Note that this relationship is not true for treatment plans only optimized for coverage as Fig. 7b shows. This further suggests that a CNN trained for generating efficient candidate beams with respect to MU can improve plan quality in terms of total MU.

In general, allowing for US-robot movement during treatment decreases the number of blocked beams because the robot can move out of the way of beams which would otherwise be blocked without changing the end effector pose. Also considering Table 1, the weighted versions of the objective function can lead to significant relative improvements with 3 LIFT angles. This indicates that the more continuous nature of the weighted objective functions (BRW, BCW) can be optimized more effectively than the counting-based versions (BR, BW).

In our approach, viable viewports were identified based on simple ray-tracing on the CT grayscale values. For clinical implementation, a more sophisticated method for generating realistic US images from CT volumes, as has been shown recently [25], could be used to select viewports in practice. However, our approach for setup optimization could then still be applied as presented in this work.

## Conclusion

We have presented an approach combining CNN-based beam metrics with heuristic search to solve the combinatorial problem of optimizing US-robot setup and treatment plan. While robotic US guidance can negatively impact treatment plan quality by blocking beams, we have shown that CNN-generated candidate beams can guide the US-robot optimization to compensate for this negative impact. We have also shown that CNN-generated candidate beams can provide a decrease in total MU when full coverage can be achieved. While the plan quality is comparable using our approach, plans without US robot are still superior to those which consider the US robot.
